# Effects of hysteroscopic septum incision versus expectant management on IVF outcomes in women with complete septate uterus: a retrospective study

**DOI:** 10.1186/s12905-024-03022-1

**Published:** 2024-03-30

**Authors:** Jiajia Zhang, Jia Kang, Xueling Song, Shuo Yang, Yan Yang, Jie Qiao, Caihong Ma

**Affiliations:** 1 Center for Reproductive Medicine, Department of Obstetrics and Gynecology, Peking University Third Hospital, Beijing, 100191 China; 2 National Clinical Research Center for Obstetrics and Gynecology, Peking University Third Hospital, Beijing, China; 3 Key Laboratory of Assisted Reproduction (Peking University), Ministry of Education, Beijing, China; 4 Beijing Key Laboratory of Reproductive Endocrinology and Assisted Reproductive Technology, Beijing, China; 5 Beijing Advanced Innovation Center for Genomics, Beijing, China; 6 Peking-Tsinghua Center for Life Sciences, Peking University, Beijing, China

**Keywords:** Septate uterus, Septum incision, In vitro Fertilization

## Abstract

**Objective:**

This retrospective study aimed to assess the impact of hysteroscopic septum incision on in vitro fertilization (IVF) outcomes among infertile women diagnosed with a complete septate uterus and no history of recurrent pregnancy loss.

**Methods:**

The study was conducted at a tertiary reproductive center affiliated with a university hospital and involved 78 women with a complete septate uterus. Among them, 34 women underwent hysteroscopic septum incision, while 44 women opted for expectant management. The primary outcome measure was the live birth rate, while secondary outcomes included clinical pregnancy rate, preterm birth rate, miscarriage rate, and ongoing pregnancy rate.

**Results:**

Women who underwent hysteroscopic septum incision demonstrated a comparable likelihood of achieving a live birth compared to those managed expectantly (25% vs. 25%, Relative Risk (RR): 1.000, 95% Confidence Interval (CI): 0.822 to 1.216). No preterm births occurred in either group. The clinical pregnancy rate, ongoing pregnancy rate, and miscarriage rate showed no significant differences between the surgical group and the expectant management group. Subgroup analyses based on the type of embryo transferred also revealed no significant differences in outcomes.

**Conclusions:**

Hysteroscopic septum incision does not appear to yield improved IVF outcomes compared to expectant management in infertile women with a complete septate uterus and no history of recurrent pregnancy loss.

**Supplementary Information:**

The online version contains supplementary material available at 10.1186/s12905-024-03022-1.

## Introduction

Septate uterus constitutes the most common congenital uterine malformation characterized by partial or total division of the uterine cavity, with an estimated incidence of 2.3% in unselected populations [1]. The American Society for Reproductive Medicine (ASRM) delineates a septate uterus as one exhibiting an indentation depth exceeding 1 cm and an indentation angle less than 90° [2]. In contrast, the European Society of Human Reproduction and Embryology (ESHRE) defines a septate uterus based on an indentation-to-wall-thickness ratio greater than 50% [3].

Given the high rate of infertility, a considerable number of subfertile patients with a septate uterus seek assistance from assisted reproductive technology. Septate uterus has been associated with decreased live birth rates after IVF/ICSI, as indicated by reduced pregnancy rate and increased pregnancy loss rate [4, 5]. Evidence also suggests that a septate uterus might be linked to adverse obstetrical complications, including an increased risk of miscarriage, preterm delivery, fetal malpresentation and intrauterine growth restriction [6, 7].

Standard treatment for septate uterus typically involves hysteroscopic resection to improve obstetrical outcomes, although its effectiveness on pregnancy results remains uncertain. Observational data support the beneficial effect of hysteroscopic septum incision, particularly for women with recurrent pregnancy loss. The meta-analysis and systematic review demonstrated a reduction in the risk of miscarriage following hysteroscopic metroplasty of the septum [8, 9]. However, a recent multicenter randomized controlled trial showed that hysteroscopic septum incision does not increase the live birth rate compared to expectant management [10]. Previous studies have reported conflicting findings regarding the effects of hysteroscopic septum incision on pregnancy outcomes in women with septate uterus following IVF. Some studies suggest a reduction in the miscarriage rate without affecting the live birth rate after IVF/ICSI in women with primary infertility [11], while others indicate increased clinical pregnancy and delivery rates after frozen embryo transfer in women with secondary infertility but no history of recurrent miscarriage [12]. However, variations in operation protocols and patient characteristics, including confusion between patients with incomplete septate uterus and those with complete septate uterus, have led to inconclusive findings.

In this retrospective study, we aimed to investigate the impact of hysteroscopic septum incision on IVF-ET outcomes in infertile women with complete septate uterus and without previous recurrent pregnancy loss or preterm birth.

## Materials and methods

### Patient recruitment

Patients were recruited retrospectively between January 2016 and June 2021 during their initial assessment for subfertility, defined as failure to achieve pregnancy within 12 months in women younger than 35 years or within 6 months in women older than 35 years. Clinical information of the patients was collected and recorded.

### Inclusion criteria

Patients were included in the analysis if they met the following criteria: (1) had complete septate uterus (2) were aged less than 40 years; and (3) were scheduled to undergo IVF/ICSI-ET/FET treatment because of fallopian tube factors, polycystic ovary syndrome (PCOS), endometriosis and/or male factors.

### Exclusion criteria

Patients with one or more of the following criteria were excluded: (1) had a history of recurrent pregnancy loss or preterm birth; recurrent spontaneous miscarriage was defined as two or more spontaneous pregnancy losses prior to 28 weeks, including biochemical gestation according to the guidelines of the Chinese Society of Obstetrics and Gynecology; (2) were preparing for preimplantation genetic test or preimplantation genetic diagnosis; and (3) had a history of endometrial lesions, tuberculosis, or intrauterine adhesion.

All individuals were informed about the option to undergo hysteroscopic septum incision, with details provided about the nature of the condition, the surgical procedure, potential outcomes, associated risks and alternative options associated with the surgery. The decision to proceed with surgery was ultimately vested in the patient, following a period of reflection and consideration of alternative management strategies, if applicable.

### Ethics declaration

This study was approved and guided by the ethical committee of the Peking University Third Hospital (project: IRB00006761-M2020004). Informed consent was obtained from all participants. All procedures performed in studies involving human participants were in accordance with the ethical standards of the institutional committee and with the 1964 Helsinki declaration and its later amendments or comparable ethical standards.

### Diagnosis of uterine anomalies

Transvaginal three-dimensional (3D) ultrasound was performed to confirm the diagnosis of a uterine anomaly. Uterine malformations were diagnosed based on the classification system originally proposed by the American Society of Reproductive Medicine and subsequently modified according to 3D ultrasound landmarks [13]. A complete septate uterus was diagnosed when a septum that completely divided the cavity from the fundus to the cervix was demonstrated on the coronal plane, with the central point of the septum at an acute angle (< 90 degrees) and uniform external convexity or with an indentation < 10 mm [13]. In addition, all of the women underwent hysteroscopy and were confirmed to have a complete septate uterus.

### Hysteroscopic uterine septum incision

After cervical dilation, the septal tissue was cut horizontally to the bottom of the uterus by using a monopolar electrode to connect the left and right uterine cavities. A Foley catheter balloon was then placed to prevent adhesion and removed 7 days postoperatively. 3D ultrasound was performed to evaluate the length of the residual septum. If the endometrial septum length was > 1 cm with the leading edge of the septum having an angle of < 90 degrees, a second operation was performed to resect the residual septum. Hysteroscopy was performed 2 months after septum incision to detect any intrauterine adhesions, endometritis, or any other abnormalities. If the uterine cavity was considered normal with good endometrial coverage, the patients underwent IVF-ET.

### Outcome measures

The primary outcome measure was live birth, defined as the birth of a living fetus beyond 24 weeks of gestational age. Secondary outcomes included the following: (1) Clinical pregnancy: a pregnancy diagnosed by the ultrasonographic visualization of one or more gestational sacs, including ectopic pregnancy; (2) Ectopic pregnancy: a pregnancy in which implantation took place outside the uterine cavity; (3) Preterm birth: a live birth or stillbirth that took place after at least 28 but before 37 completed weeks of gestation; and (4) Miscarriage: the spontaneous loss of a clinical pregnancy before 27 completed weeks of gestation or, if gestational age is unknown, the loss of an embryo/fetus of less than 1000 g. Miscarriage was divided into early miscarriage and late miscarriage. Early miscarriage referred to miscarriage occurring before 13 completed weeks; late miscarriage referred to miscarriage occurring after 13 weeks. The definitions the gestational weeks for live birth, miscarriage, and preterm birth are based on the consensus of Chinese experts [14–16].

### Statistical processing

All statistical data were analyzed using the SPSS package (SPSS 22.0, IBM Corp., USA). Continuous data are expressed as mean ± standard deviation or median (interquartile range). Count data are presented as frequency (percentage %). The statistical significance of the categorical variables was evaluated using Pearson’s χ^2^ test or Fisher’s exact test, where appropriate. For continuous data, the Shapiro-Wilk test was used to check the normality of data distribution, and the Levene test was employed for homogeneity of variance assessment. When both datasets conformed to normal distribution and homogeneity of variance, a student’s *t*-test was used for comparison. Otherwise, non-parametric test was applied. Multivariate log binomial regression was used to evaluate primary and secondary outcomes. All models were adjusted for age, body mass index (BMI), baseline serum follicle-stimulating hormone (FSH) levels, the number of embryos transferred and endometrial thickness. Among these, the number of transferred embryos is a categorical variable, while the others are continuous variables. The Hosmer-Lemeshow goodness-of-fit statistics was used to assess the fitness of the regression model in our multivariate log binomial analysis. Statistical significance was set at a p value < 0.05 (two-sided).

## Results

**Fig. 1:**
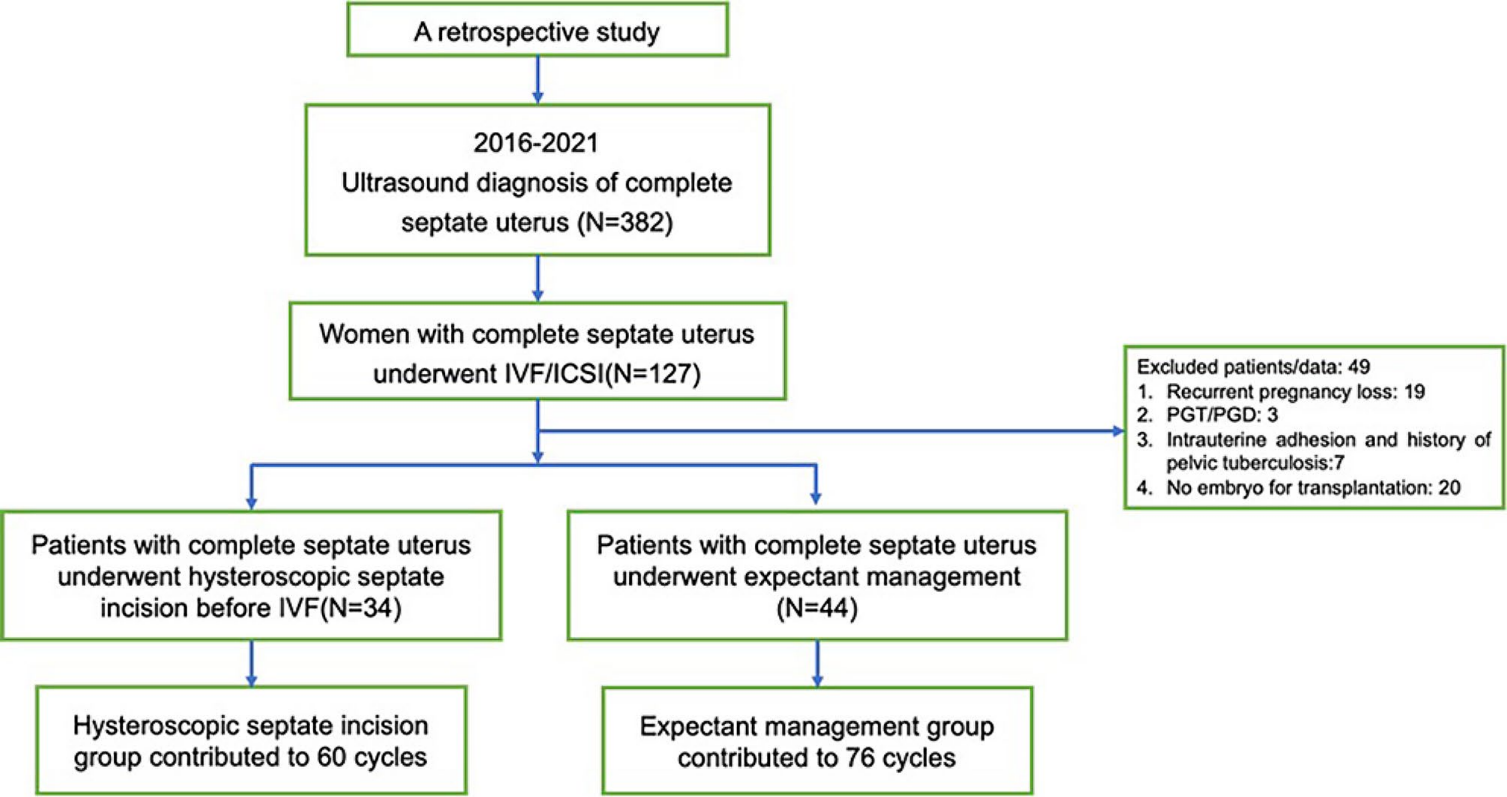
Flow chart of the study

The final cohort included 78 women with complete septate uterus. Among these women, 34 women underwent hysteroscopic septum incision and 60 embryo transfer cycles, and the 44 women underwent expectant treatment and 76 embryo transfer cycles (Fig. 1).

### Baseline characteristics

The baseline characteristics of these two groups are summarized in Table 1. The average age in the hysteroscopic septum incision group was 32.3 ± 3.2 years old, which was comparable with that in the expectant treatment group (31.7 ± 3.8 years old). Age, infertility type, infertility years, BMI, FSH levels, luteinizing hormone (LH) levels, estradiol levels and anti-Müllerian hormone (AMH) levels were comparable between the two groups (*P* > 0.05).

**Table 1:** Patient characteristics at baseline

	Hysteroscopic septum incision	Expectant management	P value
No. of patients	34	44	
Age (years) ^*^	32.3 ± 3.2	31.7 ± 3.8	0.233
**Infertility type** ^**£**^			0.765
Primary infertility	25 (73.5%)	31 (70.5%)	
Secondary infertility	9 (26.6%)	13 (29.5%)	
Infertility duration (years) ^*^	3.6 ± 1.9	3.6 ± 2.5	0.533
BMI (kg/m^2^) ^*^	23.94 ± 4.12	22.42 ± 3.36	0.113
Baseline serum FSH (IU/ml) ^*^	6.32 ± 1.92	6.81 ± 2.19	0.348
Baseline serum LH (IU/ml) ^*^	5.65 ± 3.63	4.79 ± 3.94	0.487
Baseline serum E2 (pmol/ml) ^*^	169.35 ± 55.94	158.67 ± 64.48	0.559
AMH (ng/ml) ^*^	4.49 ± 3.68	3.67 ± 2.41	0.116
**Cause of infertility** ^**£**^			0.800
Unexplained reason	4(11.8%)	3(6.8%)	
Male factor	9(26.5%)	15(34.1%)	
Tubal factor	12(35.3%)	17(38.6%)	
PCOS	8(23.5%)	7(15.9%)	
Endometriosis	1(2.9%)	2(4.5%)	

Abbreviations: BMI: Body Mass Index; FSH: Follicle stimulating hormone; LH: luteinizing hormone; E2: estradiol; AMH: Anti-Müllerian Hormone; PCOS: Polycystic Ovary Syndrome

^*^Data were presented as mean ± standard deviation

^£^Data were presented as frequency(percentage)

The surgical group underwent 60 embryo transfer cycles, and the expectant treatment group underwent 76 embryo transfer cycles. The clinical characteristics and cycle parameters in these two groups are summarized in Table 2. The surgical group was significantly older (32.7 ± 3.4 vs. 31.4 ± 3.5 years, *P* = 0.028), had a higher BMI level (24.64 ± 3.46 vs. 23.22 ± 4.21, *P* = 0.011) and a higher AMH level compared to the expectant treatment group. In the surgical group, the 60 embryo transfer cycles corporate 22 (36.7%) fresh embryo transfer cycles and 38 (63.3%) frozen-thawed embryo transfer cycles. There were 33 (43.3%) fresh cycles and 43 (56.5%) frozen-thawed cycles in the expectant management group.

**Table 2:** Clinical characteristics and cycle parameters in patients with complete septate uterus

	Hysteroscopic septum incision		Expectant management	P value	
No. of cycles	60	76			
Age (years old)^*^	32.7 ± 3.4	31.4 ± 3.52		0.028	
BMI (kg/m^2^)^*^	24.64 ± 3.46	23.22 ± 4.21		0.011	
Baseline serum FSH (IU/ml)^*^	6.42 ± 1.96	6.92 ± 2.32		0.201	
Baseline serum LH (IU/ml)^*^	5.66 ± 4.12	5.04 ± 3.67		0.724	
Baseline serum E2 (pmol/ml)^*^	160.41 ± 50.5	166.5 ± 59.9		0.820	
AMH (ng/ml)^#^	4.95 (2.25, 6.54)	2.84 (1.41, 5.50)		0.029	
**Embryo transfer cycles** ^**£**^				0.425	
Fresh cycle	22 (36.7%)	33(43.3%)			
Frozen-thawed cycle	38 (63.3%)	43(56.6%)			
Thickness of endometrium (mm)^#^	10 (9,11)	10 (9,11)		0.259	
**Type of embryos/blastocysts transferred** ^**£**^				0.188	
Single cleavage embryo	6 (10%)	8 (10.5%)			
Double cleavage embryos	28 (46.7%)	31 (40.8%)			
Single blastocyst	20 (33.3%)	35 (46.1%)			
Double blastocysts	6 (10%)	2 (2.6%)			
Mean number of transferred embryos^#^	2 (1,2)	1 (1,2)		0.126	

Abbreviations: BMI: Body Mass Index; FSH: Follicle stimulating hormone; LH: luteinizing hormone; E2: estradiol; AMH: Anti-Müllerian Hormone; PCOS: Polycystic Ovary Syndrome

*Data were presented as mean ± standard deviation

^#^ Data were presented as median (interquartile range)

^**£**^Data were presented as frequency(percentage)

### Comparison of the reproductive outcomes between the surgical group and the expectant management group

All hysteroscopic septate incisions were performed smoothly without complications such as uterine perforation or water intoxication. Three-dimensional ultrasonography was performed 2 months after hysteroscopic septate incision and revealed residual septum in two women, who subsequently underwent a second hysteroscopic septate incision. Hysteroscopy was performed 2 months after the hysteroscopic septate incision, and no intrauterine adhesions were found.

Pregnancy outcomes were assessed based on the embryo transfer cycle. Among the 60 embryo transfer cycles in the surgical group, there were 26 cycles resulting in clinical pregnancies, 8 cycles with first-trimester miscarriages, 2 cycles with ectopic pregnancies, and 15 cycles resulting in live births, all occurring at term. In the surgical group, one of the two women with residual septa did not conceive post-operation, while the other underwent cesarean section and delivered at full term after frozen-thawed embryo transfer. In the expectant management group, consisting of 76 embryo transfer cycles, there were 36 cycles with clinical pregnancies, 8 cycles with first-trimester miscarriages, and 19 cycles resulting in live births. No premature births were observed in this group.

Comparison between women undergoing hysteroscopic septum incision and those under expectant management revealed similar chances of live birth (25% vs. 25%, RR: 1.000, 95% CI: 0.822 to 1.216). Additionally, the clinical pregnancy rate in the surgical group did not significantly differ from that of the expectant group (43.3% vs. 47.4%, RR: 0.993, 95% CI: 0.684 to 1.263). Similarly, the ongoing pregnancy rate in the surgical group (26.7%) was comparable to that of the expectant group (26.7% vs. 36.8%, RR: 0.861, 95% CI: 0.684 to 1.083).

Pregnancy loss in the surgical group totaled 8 cases, including 2 mid-trimester and 6 first-trimester losses, whereas all 8 pregnancy losses in the expectant group occurred in the first trimester. Due to the limited sample size, a combined analysis of first and mid-trimester pregnancy losses was conducted to preserve statistical power. Hysteroscopic septum incision did not result in a decreased miscarriage rate compared to expectant management in the surgical group (30.8% vs. 22.2%, RR: 1.123, 95% CI: 0.824 to 1.532) (Table 3).

**Table 3:** Reproductive outcomes of the septum resection group and expectant management group

	Septum resection (*N* = 60)	Expectant management (*N* = 76)	P value	RR (95% CI)	^a^P value
Clinical pregnancy	26 (43.3%)	36 (47.4%)	0.639	0.993 (0.684,1.263)	0.638
Miscarriage	8 (30.8%)	8 (22.2%)	0.448	1.123 (0.824, 1.532)	0.462
Ongoing pregnancy	16 (26.7%)	28 (36.8%)	0.208	0.861 (0.684,1.083)	0.202
Live birth	15 (25%)	19 (25%)	1.000	1.000 (0.822, 1.216)	1.000

^a^Adjusted for age, BMI, FSH level, type of embryos/blastocysts transferred, number of embryos transferred and endometrial thickness

Subgroup analysis by type of embryo transferred is shown in Table 4. In a total of 73 cycles, cleavage stage embryos were transferred, and blastocysts were transferred in 63 cycles. The live birth rate, clinical pregnancy rate, ongoing pregnancy rate and miscarriage rate were similar between the two groups when either cleavage-stage embryos or blastocysts were transferred (Table 4).

**Table 4:** Subgroup analysis of reproductive outcomes stratified by the type of embryo transferred

	Septum incision	Expectant management	P value	RR (95% CI)	^a^P value
**Cleavage embryo**	**(** ***N*** **( = 34)**	**(** ***N*** ** = 39)**			
Clinical pregnancy	14 (41.2%)	17 (43.6%)	0.835	0.959 (0.647, 1.422)	0.835
Miscarriage	2 (14.3%%)	3 (17.6%)	1.000	0.397 (0.025,6.200)	0.510
Ongoing pregnancy	10 (29.4%)	14 (35.9%)	0.556	0.908 (0.660,1.250)	0.555
Live birth	9 (26.5%)	9 (23.1%)	0.737	1.046 (0.803, 1.364)	0.739
**Blastocyst**	**(** ***N*** ** = 26)**	**(** ***N*** ** = 37)**			
Clinical pregnancy	12 (46.2%)	19 (51.4%)	0.685	0.903 (0.556, 1.469)	0.682
Miscarriage	6 (50%)	5 (26.3%)	0.339	1.474 (0.788, 2.757)	0.225
Ongoing pregnancy	6 (23.1%)	14 (37.8%)	0.215	0.808 (0.582,1.122)	0.203
Live birth	6 (23.1%)	10 (27.0%)	0.727	0.949 (0.711,1.265)	0.720

^a^Adjusted for age, BMI, FSH level, number of embryos transferred and endometrial thickness

## Discussion

This retrospective study found that there was no statistically significant difference in clinical pregnancy rate, ongoing pregnancy rate, or live birth rate between the surgical and expectant management groups. Moreover, irrespective of the type of embryo transferred, these rates remained similar between the two groups per transfer cycle.

### Interpretations

Previous retrospective investigations, meta-analyses, and systematic reviews have suggested that hysteroscopic septum resection may reduce miscarriage rates and offer potential benefits, particularly for women with a septate uterus experiencing recurrent miscarriages [8, 17, 18]. However, its influence on live birth rates, clinical pregnancy rates, or preterm delivery rates appears inconclusive [8]. Among these, two studies exclusively focused on women with uterine septum and primary infertility. One study found that septum resection did not significantly affect live birth rates [11], while another reported comparable IVF outcomes between septum resection and normally shaped uteri [19]. Notably, our study focused on women with a complete uterine septum who did not experience recurrent miscarriages, a subgroup with limited literature documentation. Our findings align with those observed in women experiencing primary infertility, indicating that hysteroscopic septum resection may not confer benefits to IVF outcomes in this population.

However, our results indicated a slightly higher miscarriage rate in the surgical group compared to the expectant group, albeit without statistical significance[30.8% vs. 22.2%, RR: 1.123, 95% CI (0.824, 1.532)]. This unexpected result may be attributed to the limited sample size and other factors influencing post-transplant miscarriage rates. Additionally, the potential impact of surgical incision on the endometrium versus the benefits of improving uterine cavity morphology through septum resection remains unclear.

Regarding postoperative complications, none were observed in our study. This could be attributed to the small sample size, making it difficult to detect intrauterine adhesions. Moreover, the hysteroscopic uterine septum incisions were performed by experienced reproductive gynecologists, minimizing trauma to healthy tissues. Additional strategies to prevent intrauterine adhesions include postoperative placement of intrauterine balloon catheters for 5–7 days and a course of postoperative conjugated estrogen.

### Strengths and limitations

While previous studies have extensively examined the impact of septum resection on pregnancy outcomes, our study’s novelty lies in its focus on women with no history of recurrent pregnancy loss. However, limitations include the relatively small sample size and inherent biases in retrospective studies. Future well-designed randomized trials with larger sample sizes are needed to compare IVF outcomes in patients with complete septate uteri after surgical correction versus those without correction.

### Implications for practice and research

Hysteroscopic septum resection is commonly performed worldwide to improve reproductive outcomes in women with a septate uterus. However, our results suggest that it may not offer significant advantages over expectant management in women with a complete septate uterus and no previous adverse pregnancy outcomes before IVF/ICSI. Further randomized controlled trials are warranted to inform the use of hysteroscopic surgery in this population.

## Conclusion

In conclusion, while hysteroscopic septum incision is a well-established method to correct uterine malformations, it may not improve IVF-ET outcomes in infertile women with a complete septate uterus and no history of recurrent pregnancy loss or preterm delivery. Patients should receive comprehensive information regarding the advantages and disadvantages of the operation and undergo individualized evaluations in clinical practice.

### Electronic supplementary material

Below is the link to the electronic supplementary material.

Supplementary Material 1

## Data Availability

Researchers who want access to datasets for replication should apply to kangjiasdu@163.com.

## Abbreviations

AFC: Antral follicle count
AFS: American Fertility Society
ART: Assisted reproduction technology
BMI: Body Mass Index
OS: Ovarian stimulation
ESHRE: European Society of Human Reproduction and Embryology
ESGE: European Society for Gynecological Endoscopy
ET: Embryo transfer
FET: Frozen thawed embryo transfer
FSH: Follicle stimulating hormone
ICSI: Intracytoplasmic sperm injection
IVF: In vitro fertilization
TRUST: the randomized uterine septum transection trial
2D: Two-dimensional
3D: Three-dimensional
