# Supplementary material for: Effects of hysteroscopic septum incision versus expectant management on IVF outcomes in women with complete septate uterus: a retrospective study
Source: BMC Womens Health. 2024 Mar 30;24:202. doi: 10.1186/s12905-024-03022-1 (PMC10981310; doi:10.1186/s12905-024-03022-1)
Supplement: Supplementary file 1 — Supplementary Material 1 [file 12905_2024_3022_MOESM1_ESM.docx]

## Ovarian stimulation protocol

Different ovarian stimulation protocols were adopted according to the patient’s situation. All women who underwent IVF/ICSI cycles received controlled ovarian hyperstimulation protocols as previously described(1, 2). Briefly, the downregulation protocol included the gonadotrophin-releasing hormone (GnRH) agonist long protocol and the ultralong protocol. In the long protocol, patients underwent pituitary downregulation by midluteal administration of a GnRH agonist, daily injections of 0.1 mg triptorelin acetate, or a single injection of 1.3 mg/1.8 mg triptorelin followed by a hormone test 14 days later. In the GnRH agonist ultralong protocol, patients underwent ovarian stimulation that was initially by the recombinant FSH (rFSH) protocol initial between Day 28 and Day 30 of their menstrual cycle following pituitary downregulation by 3.75 mg of triptorelin acetate or leuprorelin acetate on the first day of the cycle. In the antagonist protocol, the GnRH antagonist (ganirelix acetate or cetrorelix acetate) was given beginning from Day 5 of rFSH injection, or when the leading follicle reached 14 mm, to the day of human chorionic gonadotrophin (hCG) administration. In the agonist protocol, patients were administered a GnRH agonist from the second day of their menstrual cycle onward. Ovarian stimulation (OS) was given to patients receiving rFSH or human menopausal gonadotropin (HMG) in different flexible protocols. In the micro-stimulation protocol, 2.5 mg letrozole or 50 mg clomiphene citrate was administered daily from Day 2 to 6 of menstruation, and 150 IU of recombinant FSH was initiated from Day 5.

In all treatment protocols, once at least two leading follicles reached a size of ≥18 mm, 5000 to 10 000 IU hCG or 250 μg recombinant hCG (r-hCG) was administered to trigger ovulation, and ovum collection was performed between 36 and 38 hours later. Oocytes were fertilized by either conventional IVF or intracytoplasmic sperm injection. On the same day, semen was collected, and oocytes were cultured for 2–6 hours, followed by fertilization, which was observed for 16–18 hours.

The aforementioned ovulation induction protocol is equally applicable to both fresh embryo transfer and frozen-thawed embryo transfer cycles.

## Endometrial preparation for embryo transfer and luteal support

Embryo transfer was accomplished on Days 3 or 5 of the cycle according to the Code of Practice for Assisted Reproductive Technology developed by the Ministry of Health of the People's Republic of China. High-quality embryos were defined as D3 embryos that had more than 7 blastomeres and had fragmentation less than 10%. High-quality blastocysts are typically defined as those reaching stage 3 or beyond in Gardner's grading system, with inner cell mass and trophectoderm scores excluding a C grade(3).

In the fresh embryo transfer cycle, subsequent to the transfer procedure, patients were administered 90 mg of vaginal progesterone gel, complemented by a daily dosage of 20 mg of dydrogesterone for luteal support.

Endometrial preparation within the frozen cycle encompassed either a natural or artificial protocol, which was executed in accordance with the diagnostic and therapeutic standards of our institution. In the natural cycle or ovulation induction regimen, didroxyprogesterone at a dosage of 20–40 mg/day was initiated on the day of transplantation. Alternatively, the artificial cycle commenced between days 1–3 of menstruation, involving oral progesterone at a dose of 6 mg/day. Ultrasound monitoring of endometrial status occurred after 10 days of medication intake. Progesterone conversion (administering progesterone at 40 mg/day or vaginal progesterone gel at 90 mg/day) was undertaken post-achievement of an endometrial thickness of 8 mm, designated as D0. Embryo transfer was conducted on either D3 or D5.

On the 30th day after embryo transfer, transvaginal ultrasound was performed to confirm the intrauterine pregnancy with a live fetus, and the luteal support drug dose was gradually decreased for discontinuation.

1. Wang H, Gao H, Chi H, Zeng L, Xiao W, Wang Y, et al. Effect of Levothyroxine on Miscarriage Among Women With Normal Thyroid Function and Thyroid Autoimmunity Undergoing In Vitro Fertilization and Embryo Transfer: A Randomized Clinical Trial. JAMA. 2017 Dec 12;318(22):2190-8.

2. Yang R, Niu ZR, Chen LX, Liu P, Li R, Qiao J. Analysis of related factors affecting cumulative live birth rates of the first ovarian hyperstimulation in vitro fertilization or intracytoplasmic sperm injection cycle: a population-based study from 17,978 women in China. Chin Med J (Engl). 2021 Jun 4;134(12):1405-15.

3. Embryology ESIGo, Alpha Scientists in Reproductive M. The Vienna consensus: report of an expert meeting on the development of art laboratory performance indicators. Hum Reprod Open. 2017;2017(2):hox011.
